# Developmental malformations in Huntington disease: neuropathologic evidence of focal neuronal migration defects in a subset of adult brains

**DOI:** 10.1007/s00401-021-02269-4

**Published:** 2021-01-30

**Authors:** R. A. Hickman, P. L. Faust, M. K. Rosenblum, K. Marder, M. F. Mehler, J. P. Vonsattel

**Affiliations:** 1 Department of Pathology and Cell Biology, Columbia University Irving Medical Center and New York Presbyterian Hospital, New York, USA; 2 Department of Pathology, Memorial Sloan Kettering Cancer Center, New York, USA; 3 Department of Neurology and Psychiatry, Columbia University Irving Medical Center, New York, USA; 4 Taub Institute for Research on Alzheimer’s disease and the Aging Brain, Columbia University Medical Center, 710 West 168th Street, New York, NY 10032 USA; 5 The Saul R. Korey Department of Neurology, Albert Einstein College of Medicine, New York, USA; 6 Dominick P. Purpura Department of Neuroscience, Albert Einstein College of Medicine, New York, USA; 7 Department of Psychiatry and Behavioral Sciences, Albert Einstein College of Medicine, New York, USA

**Keywords:** Huntington disease, Heterotopia, Malformation, Hamartoma, Development, Huntingtin

## Abstract

Neuropathologic hallmarks of Huntington Disease (HD) include the progressive neurodegeneration of the striatum and the presence of Huntingtin (HTT) aggregates that result from abnormal polyQ expansion of the *HTT* gene. Whether the pathogenic trinucleotide repeat expansion of the *HTT* gene causes neurodevelopmental abnormalities has garnered attention in both murine and human studies; however, documentation of discrete malformations in autopsy brains of HD individuals has yet to be described. We retrospectively searched the New York Brain Bank (discovery cohort) and an independent cohort (validation cohort) to determine whether developmental malformations are more frequently detected in HD versus non-HD brains and to document their neuropathologic features. One-hundred and thirty HD and 1600 non-HD whole brains were included in the discovery cohort and 720 HD and 1989 non-HD half brains were assessed in the validation cohort. Cases with developmental malformations were found at 6.4–8.2 times greater frequency in HD than in non-HD brains (discovery cohort: OR 8.68, 95% CI 3.48–21.63, *P*=4.8 × 10^-5^; validation cohort: OR 6.50, 95% CI 1.83–23.17, *P*=0.0050). Periventricular nodular heterotopias (PNH) were the most frequent malformations and contained HTT and p62 aggregates analogous to the cortex, whereas cortical malformations with immature neuronal populations did not harbor such inclusions. HD individuals with malformations had heterozygous *HTT* CAG expansions between 40 and 52 repeats, were more frequently women, and all were asymmetric and focal, aside from one midline hypothalamic hamartoma. Using two independent brain bank cohorts, this large neuropathologic series demonstrates an increased occurrence of developmental malformations in HD brains. Since pathogenic *HTT* gene expansion is associated with genomic instability, one possible explanation is that neuronal precursors are more susceptible to somatic mutation of genes involved in cortical migration. Our findings further support emerging evidence that pathogenic trinucleotide repeat expansions of the *HTT* gene may impact neurodevelopment.

**Supplementary Information:**

The online version contains supplementary material available at 10.1007/s00401-021-02269-4.

## Introduction

Increasing attention has been afforded to the neurodevelopmental abnormalities in Huntington Disease (HD) and its possible contributions to neurodegeneration [3, 10, 41, 42, 47]. The pathogenic CAG repeat expansion in the *HTT* gene that causes HD exists from conception and continues to be expressed throughout development and the remainder of life [11, 38]. Healthy development depends on the gene’s encoded protein, Huntingtin (HTT), which has vital roles in gastrulation and corticogenesis [6, 16, 25, 64]. Homozygous *HTT* knockout mice cannot develop beyond mid-embryonic stages (E7.5-8.5) and *HTT* hypomorphic mice exhibit neuronal migration defects with later striatal degeneration [5, 20, 45]. Limited data in humans suggest that there are developmental differences in gene-expanded carriers (GEC). The rate of brain growth is reduced in GEC and there is a tendency for smaller intracranial volumes when compared to age-matched control subjects [35, 47, 60]. Furthermore, brains of human fetal GEC have wide-ranging anomalies in corticogenesis. At 13 weeks gestational age, HTT and junctional complex proteins are mislocalized at the ventricular zone, and neural progenitor cells exhibit various defects, including abnormalities in cellular polarity, differentiation, and ciliogenesis [10].

However, despite these emerging data, there has been no reported evidence of malformations due to either patterning defects or neuronal migration disorders in adult human post-mortem HD brains. While the exact frequency of cortical malformations in the general population is unknown, they are considered to be rare; the most commonly detected malformation are periventricular nodular heterotopias (PNH) [4, 8, 52]. Gray matter heterotopias are characterized by abnormally located islands of gray matter within white matter that are the result of aborted neuronal migration during cortical development [9]. Although infrequent, they have been reported in large neurodegenerative brain bank cohorts and can share the proteinopathies found within the cortex [12, 31].

We hypothesized that malformations are more frequent in brains of individuals with HD than those without. We searched the database at the New York Brain Bank (NYBB) between 2001 and 2020 for malformations and reviewed their neuropathologic characteristics. The standardized brain bank protocol used at the NYBB permits consistent comparisons across neurodegenerative diseases and controls without otherwise introducing sampling biases across brains. We find that malformations are more frequent in HD brains than non-HD brains at the NYBB and this observation is replicated in another larger autopsy cohort. Similar to other neurodegenerative diseases with distinctive proteinopathies, HTT and p62 aggregates can coexist within the cerebral cortex and heterotopias. The implications of this increased occurrence of developmental anomalies in HD and their relationship to the expanded *HTT* gene are discussed.

## Materials and methods

### Study subjects and dissection protocol

The majority of brains included in the discovery cohort (NYBB) were processed using the standardized protocol as outlined previously [62]. Most fresh brains were divided by a sagittal cut through the corpus callosum and brainstem resulting in two half brains; one half brain would then be processed for banking of fresh-frozen samples for research. A pathologist macroscopically examined all fresh brain slices. The non-banked half or whole brain was then fixed by immersion in 10% neutral-buffered formalin solution for 2 weeks and then evaluated as per the NYBB protocol. Briefly, the cerebral hemispheres were sectioned in the coronal plane, the brainstem in the transverse plane, and cerebellum in the sagittal plane. All sections were performed at 0.3 cm intervals. The Brodmann map was utilized for blocking cortical regions to allow for consistent comparisons of neocortical regions across brains [14]. A standardized series of formalin-fixed tissue blocks was then processed, embedded in paraffin wax and sectioned at 7 μm thickness. The vast majority of cases were examined and diagnosed by one of the authors who has extensive expertise in neurodegenerative disease (JPV).

The validation cohort represented an independent cohort of brains that were examined by one of the authors (JPV) before 2001 at the Harvard Brain Tissue Resource Center (HBTRC) at McLean Hospital, Boston, USA. This cohort of brains often had one half brain either frozen *en bloc* or dissected by a research technician, while the other fixed half brain was macroscopically and microscopically examined by a neuropathologist.

All brain donors were consented for autopsy and brain donation. All procedures performed were in accordance with the ethical standards of the institution and with the 1964 Helsinki declaration and its later amendments. This study is considered exempt from Columbia’s institutional review board, since it utilizes only post-mortem human tissues.

### Database searches

The discovery cohort was assembled from brains banked at the NYBB between 2001 and 2020. The relational database used at the NYBB is a customized archive of data that uses the scripting language of FileMaker Pro to contain all specimens that have been studied at the NYBB [32]. Of 1766 cases, 1731 cases met the inclusion criteria of having an intact brain specimen that was macroscopically and microscopically examined. In May 2020, this database was searched using the keyword search terms, “heterotopia” and “malformation”. One case with a heterotopia was excluded, because the individual had a genetic predisposition for developmental malformations. Twenty cases were identified and histology slides were reassessed. Then, 12 cases with malformations of the following conditions were assessed in the non-HD cohort: (1) Alzheimer Disease neuropathologic changes (ADNC) with or without Lewy body-containing neurons, (2) Diffuse Lewy body disease (DLBD)/Parkinson Disease (PD) with or without ADNC, and (3) Cases without neurodegenerative disease (control). Histologic changes associated with aging, such as primary age-related tauopathy [28] or age-related vascular disease, were included in this latter group.

A second database using Microsoft Excel comprised the cohort of brains assessed by one of the authors (JPV) between 1981 and 2000 at the HBTRC. This anonymized database included age, sex, whether a half brain or whole brain was examined by a pathologist, and the primary neuropathologic diagnoses of each brain. After excluding consult cases, the database was searched for the proportion of half brains examined by a pathologist that harbored a brain malformation in HD and non-HD cases. Additionally, the non-HD groups were then subdivided into three further categories as follows: (1) Cases with ADNC with or without DLBD/PD, (2) DLB/PD with or without ADNC, and (3) Controls, defined as cases without neurodegenerative disease as outlined in the validation cohort.

### Histochemical staining protocols

Luxol-fast blue counterstained with hematoxylin and eosin sections were examined. Paraffin sections of ten cases (#1–5, #7–8, #13, #16, and #18) were immunostained on an automated immunostaining platform using a DAB with or without alkaline phosphatase dual staining system on a Roche Ventana staining platform (Table 1). Hematoxylin counterstains were performed. We used the 2B4 anti-HTT antibody that targets a.a. 1-82 of the N-terminal end of HTT; this antibody has been validated in human HD brains [18, 27]. The anti-CD34 antibody (QBEND/10 clone) targets a.a. 43-49 of the extracellular domain and is expressed in stem cells as well as endothelial cells [2].

**Table 1:** Details regarding primary immunohistochemical stains used in this study

Antibody	Company	Catalogue number	Dilution	Primary antibody incubation time (min)	Protocol	Platform	Chromagen
Huntingtin/p62 double stain	Millipore/Abcam	MAB5492/ ab207305	1:2000 each	32/24	64 min (CC1)	Roche Ventana Instrument	Brown- Huntingtin Red-p62
Huntingtin	Millipore	MAB5492	1:2000	32	64 min (CC1)		Brown
P62	Abcam	Ab207305	1:2000	24	64 min (CC1)		Brown
CD34	Leica	PA0212	Predilute	30	20 min (ER2)	Leica Bond III	Brown

### Statistical analyses

Statistical analyses were performed on GraphPad Prism version 8.4.2 and IBM SPSS® Statistics software version 27. Statistical comparisons between the demographic distribution and frequency of malformations of HD and non-HD groups were performed using two-sided Fisher’s exact tests (Supplemental Table 1). Odds ratios (OR) and 95% confidence intervals (CI) were determined using the Baptista–Pike method. For a final overall model, we applied binary logistic regression to both cohorts with malformation as the dependent variable to examine the interactions of age at death, sex, and HD status. One-hundred and thirty-one of 4049 participants lacked data for age and/ or sex (43 HD and 88 non-HD) and were therefore excluded from this analysis. Then, to explore the association between sex and age at death with malformation in HD, binary logistic regression was assessed only in HD participants using the combined cohort. Thirty-eight participants lacked data for age and/or sex and were thus excluded in this latter assessment.

Throughout this study, a *P* value < 0.05 was deemed statistically significant.

## Results

### Malformations are more frequent in HD than non-HD brains

1730 brains were included in the discovery cohort and the demographic details are provided in Supplemental Table 1. Of these, 130 brains were diagnosed as HD and 6.2% had at least one malformation that was either a periventricular nodular heterotopia (*n* = 4), multinodular and vacuolating neuronal tumor of the cerebrum (MVNT) (*n* = 1), a glioneuronal hamartoma (*n* = 1), or a cerebellar nodular heterotopia (*n *= 2). This frequency was 8.2-fold greater than that found in non-HD brains (0.75%, *n *= 12 of 1600, OR 8.68, 95% CI 3.48–21.63, *P *= 4.8 × 10^–5^, two-sided Fisher’s exact test). Aside from the midline hamartoma (case #4), all HD malformations were asymmetric and solitary. Individuals with malformations in this discovery cohort are shown in Table 2. Other malformations, such as tubers or type II focal cortical dysplasia, were not recognized.

**Table 2:** Demographics, clinical information and locations of malformations between HD versus non-HD brains of the discovery cohort

Group	Case #	Age at death (years)	Sex	CAG repeat length of expanded allele	Post-mortem diagnosis	Location	Malformation type
HD	1	41	Woman	47	HD 3/4*	Atrium, left	Periventricular nodular heterotopia
	2	68	Woman	44	HD 3/4	Roof of temporal horn of lateral ventricle, left	Periventricular nodular heterotopia
	3	58	Woman	Unknown	HD 3/4	Above tail of caudate nucleus, left	Periventricular nodular heterotopia
	4	59	Woman	42	HD 3/4	Tuber cinereum	Hypothalamic hamartoma
	5	75	Woman	42	HD 2/4	Prefrontal subcortical white matter, left	Multinodular and vacuolating neuronal tumor of the cerebrum (MVNT)
	6	72	Man	40	HD 2/4	Cerebellar white matter, left	Cerebellar nodular heterotopia
	7	72	Woman	Unknown	HD 1/4	Cerebellar white matter, left	Cerebellar nodular heterotopia
	8	36	Woman	52	HD 4/4	1) Above tail of caudate nucleus, right 2) Insular cortex, right	1) Periventricular nodular heterotopia 2) Glial microhamartoma/ heterotopia
Non-HD	9	92	Woman	N/A	ADNC	Frontal subcortical white matter, left	Periventricular nodular heterotopia
	10	91	Man		ADNC	Superolateral angle of the lateral ventricle, left	Periventricular nodular heterotopia
	11	79	Woman		ADNC	Prefrontal subcortical white matter, left	Subcortical nodular heterotopia
	12	86	Man		ADNC	Roof of temporal horn	Periventricular nodular heterotopia
	13	90	Woman		ADNC	Temporal pole, subcortical white matter, left	Subcortical band heterotopia
	14	67	Woman		ADNC	Alveus, hippocampus, left	Periventricular nodular heterotopia
	15	74	Man		Control	Frontal, right	Multiple heterotopia, nodular and band
	16	66	Man		Control	Superolateral angle of lateral ventricle, right	Periventricular nodular heterotopia
	17	90	Man		ADNC	Temporal pole, subcortical white matter, left	MVNT
	18	80	Man		DLB/ PD	Optic radiation, temporal, left	Periventricular nodular heterotopia
	19	81	Man		Multi-infarct dementia	Cerebral leptomeninges	Leptomeningeal glioneuronal heterotopias, multifocal
	20	93	Woman		ADNC	Cerebellar white matter, left	Cerebellar nodular heterotopia

^*^Refers to Vonsattel grade of HD [63]

To replicate our findings, we assessed the files of an independent cohort of 2709 half brains, of which 720 were half brains from HD individuals that were received at the HBTRC (Table 3; Supplemental Table 1). Seven of 720 (0.97%) HD half brains had heterotopias whereas only 3 of 1989 (0.15%) non-HD half brains had such malformations. Although the overall detection of malformations was approximately three times less than in the discovery cohort, heterotopias in HD were still 6.4 times more frequent in HD than non-HD half brains (OR 6.50, 95% CI 1.83–23.17, *P *= 0.0050, two-sided Fisher’s exact test), akin to that found in the discovery cohort.

**Table 3:** Demographics, clinical information, and locations of heterotopias between HD versus non-HD brains of the validation cohort.

Group	Case #	Age at death (years)	Sex	CAG repeat length of expanded allele	Post-mortem diagnosis	Location	Malformation type
HD	21	75	Man	Unknown	HD, 3/4*	Roof of temporal horn	Periventricular nodular heterotopia
	22	47	Woman	Unknown	HD, 4/4	Roof of temporal horn, right	Periventricular nodular heterotopia
	23	58	Man	Unknown	HD, 4/4	Superolateral angle of lateral ventricle, frontal, left	Periventricular nodular heterotopia
	24	69	Woman	40	HD, 4/4	Roof of temporal horn, right	Periventricular nodular heterotopia
	25	36	Woman	Unknown	HD, 1/4	Cerebellar white matter, left	Cerebellar nodular heterotopia
	26	60	Woman	Unknown	HD, 3/4	Roof of temporal horn, left	Periventricular nodular heterotopia
	27	49	Woman	Unknown	HD, 2/4	Roof of temporal horn, right	Periventricular nodular heterotopia
Non-HD	28	71	Woman	N/A	Control	Parietal white matter, right	Periventricular nodular heterotopia
	29	91	Man		ADNC	Roof of temporal horn, right	Periventricular nodular heterotopia
	30	43	Man		Hippocampal sclerosis (schizophrenia, clinical)	Roof of temporal horn, right	Periventricular nodular heterotopia

*ADNC* Alzheimer disease neuropathologic changes

*Refers to Vonsattel grade of HD [63]

Further subdivisions of malformation frequency between both cohorts are shown in Table 4. In the discovery cohort, the frequencies of malformations in HD were significantly greater than all of the other categories. In the validation cohort, the frequency of malformations was also significantly greater in HD brains than in non-HD and in ADNC brains with similar odds ratios to that of the discovery cohort. However, significance could not be achieved in the DLB/PD and control groups, probably because they were underpowered.

**Table 4:** Comparisons of malformation frequency between HD and other diseases/controls in the cohorts.

	Discovery cohort				Validation cohort			
Proportion with malformations	HD	Non-HD	*P*	OR (CI)	HD	Non-HD	*P*	OR (CI)
	8/130 (6.15%)	12/1600 (0.75%)	4.8 ×10^-5^	8.68 (3.48–21.63)	7/720 (0.97%)	3/1989 (0.15%)	0.0050	6.50 (1.83–23.17)
		ADNC	*P*	OR (CI)		ADNC	*P*	OR (CI)
		8/685 (1.17%)	0.0014	5.55 (2.06–14.88)		1/798 (0.12%)	0.031	7.83 (1.18–88.3)
		DLB/ PD	*P*	OR (CI)		DLB/ PD	*P*	OR (CI)
		1/212 (0.47%)	0.0023	13.84 (2.09–154.3)		0/ 118 (0%)	0.60	–
		Control	*P*	OR (CI)		Control	*P*	OR (CI)
		2/235 (0.85%)	0.0049	7.64 (1.84–36.03)		1/626 (0.16%)	0.075	6.14 (0.8–69.27)

In contrast to the discovery cohort, where the figures represent the total number of whole brains examined, the figures in the validation cohort represent the total number of half brains. *P* values represent two-sided Fisher’s exact tests comparing the frequency of heterotopias in HD and that of the other respective conditions.

In the discovery cohort, malformations were more frequent in women (11.5%) than in men (1.4%) with HD (OR 8.82, 95% CI 1.48–100.6, *P *= 0.026, two-sided Fisher’s exact test). This was in contrast to brains without HD where there was no significant difference between women (0.6%) and men (0.9%) with malformations (OR 0.72, 95% CI 0.26–2.04, *P *= 0.77, two-sided Fisher’s exact test). In the validation cohort, however, significance could not be achieved as to whether malformations were more frequent in women (1.7%) than men (0.63%) with HD (OR: 2.70, 95% CI 0.58–13.63, *P *= 0.27, two-sided Fisher’s exact test). There was no significant difference in frequency between the proportion of women in the HD and non-HD cohorts in either the discovery or validation cohorts (Supplementary Table 1).

Values for CAG repeat expansion in the *HTT* gene were available for 102 HD brains in the discovery cohort and the median expanded CAG allele was 45 (interquartile range: 5). In this cohort, HD brains with malformations with known CAG expansions had expanded CAG values that were typically found in the lower ranges of CAG repeat expansion (CAG: 40–52).

We next sought to model the clinicopathologic associations with malformations using binary logistic regression on both cohorts combined (Table 5). Overall, malformations were significantly associated with HD (OR 5.12, 95% CI 2.25–11.66, *P *< 0.0001), but not with age at death or sex. When only HD individuals were assessed, women significantly associated with malformations (OR 4.56, 95% CI 1.27–16.3, *P *= 0.020), whereas age at death was not significant (OR 0.99, 95% CI 0.96–1.03, *P *= 0.68). No significant difference between the age at death (OR 1.03, 95% CI 0.99–1.08, *P *= 0.17) or sex (OR 0.44, 95% CI 0.15–1.32, *P *= 0.14) was found in the non-HD participants.

**Table 5:** Associations with malformation in both cohorts

Characteristic	Odds ratio (95% CI)	*P*
All participants (HD and non-HD), *n *= 3918		
HD status	5.12 (2.25–11.66)	9.9 × 10^-5^
Age at death	1.01 (0.98–1.03)	0.60
Sex	1.37 (0.66–2.85)	0.40
HD only, *n *= 711		
Age at death	0.99 (0.96–1.03)	0.68
Sex	4.56 (1.27–16.3)	0.020
Non-HD only, *n* = 3207		
Age at death	1.03 (0.99–1.08)	0.17
Sex	0.44 (0.15–1.32)	0.14

Binary logistic regression with malformation as dependent variable. HD status was dichotomized as HD = 1, non-HD = 0 and sex as female = 1, male = 0.

### Pathologic features of malformations in the HD groups

Slides and formalin-fixed paraffin-embedded blocks of cases #1–20 of the NYBB discovery cohort were available for reassessment. Additional sections of cases #1–5, #7–8, #13, #16, and #18 were immunohistochemically stained against Huntingtin and p62.

**Figure 1:**
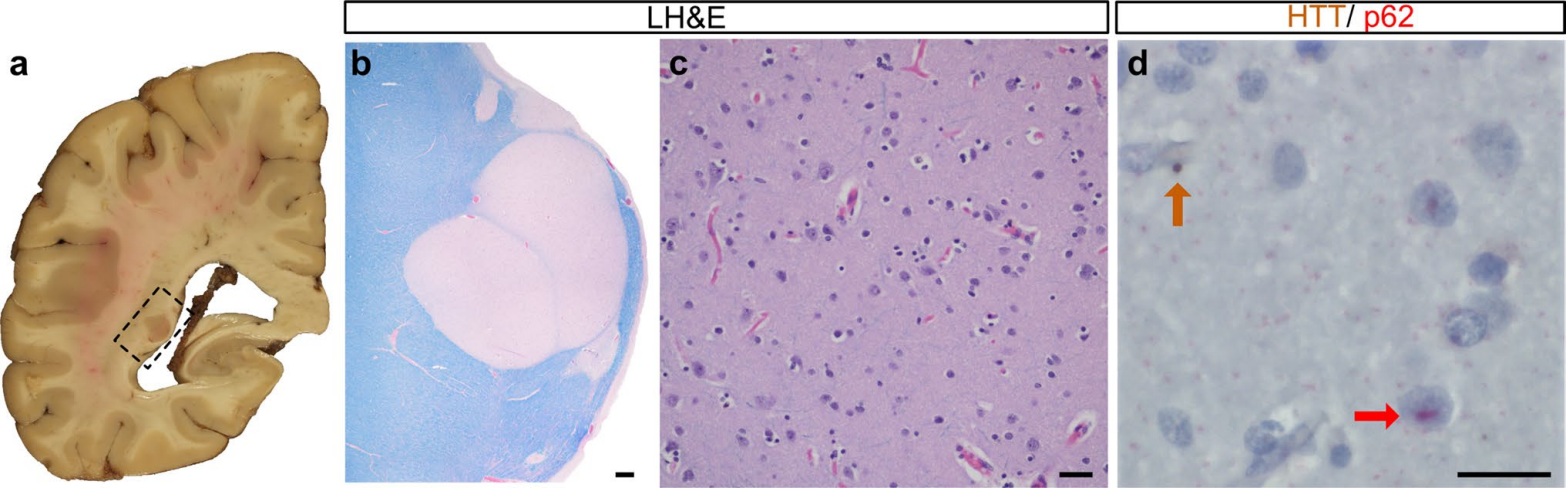
Macroscopic and microscopic photographs of a 41-year-old woman (CAG: 47/14) with a PNH (case #1). **a** The PNH in case 1 is beneath the ependymal lining of the left atrium (dashed rectangle outlines the malformation). **b** This multinodular heterotopia is surrounded by myelinated fibers. **c** The heterotopia comprises non-dysmorphic neurons with haphazardly arranged apical dendrites amongst oligodendrocytes and astrocytes. **d** This heterotopia harbored HTT and p62 aggregates. Brown arrows indicate HTT aggregates; red arrows indicate p62 aggregates. Stains: **b**, **c** LH&E; **d** HTT/p62 immunostain. Scale bars: **b** 200 µm; **c** 50 µm; **d** 20 µm.

**Figure 2:**
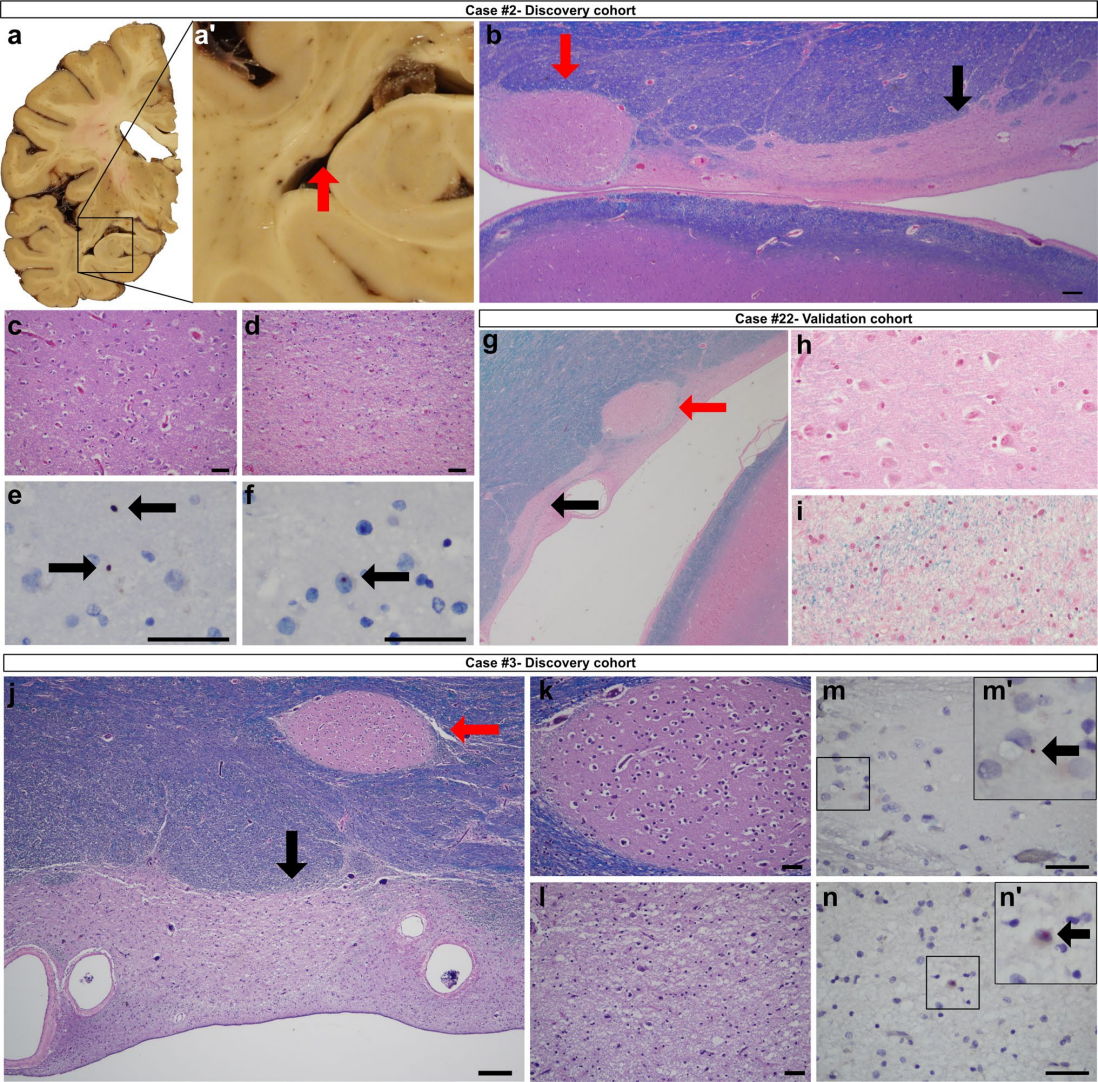
Three examples of individuals with HD (cases #2, #3, and #22) that have PNH around the temporal horn of the lateral ventricle. In all three instances, the tail of the caudate nucleus (black arrow) is severely atrophic, whilst the nearby heterotopia (red arrow) appears relatively spared of injury. **a** Coronal slice of the left cerebral hemisphere at the level of the mammillary body revealing a flattened body of the caudate nucleus with atrophy of the lenticular nucleus and a focal nodule protruding into the temporal horn of the lateral ventricle (red arrow, a’). **b** A photomicrograph of an LH&E-stained section indicating the ovoid heterotopia. The heterotopia (red arrow) is in close proximity to the atrophic tail of the caudate nucleus (black arrow). **c**, **d** In contrast to the tail of the caudate nucleus (**d**), the heterotopia (**c**) does not show neuronal loss or gliosis, but a collection of neurons and glia. The tail of the caudate nucleus is without neurons but with fibrillary gliosis. **e**, **f** Similar to the neocortex of this case (not shown), both HTT (**e**) and p62 (**f**) aggregates are found within the heterotopia. **g** In case #22, the heterotopia (red arrow) is seen adjacent to the tail of the caudate nucleus (black arrow). **h**, **i** The tail of the caudate (**i**) shows gliosis and neuronal loss in contrast to the relatively spared heterotopia (**h**). **j**–**l** In case #3, the tail of the caudate nucleus (black arrow) shows neuronal loss and fibrillary gliosis (**l**) in contrast to the oval-shaped heterotopia (**k**). **m**, **n** HTT (brown chromagen, m’ inset) and p62 aggregates (red chromagen, n’ inset) were found in the heterotopia and adjacent tail of caudate nucleus, respectively. Scale bars: **b**, **j** 200 µm; **c**–**f**, **k**–**n** 50 µm.

The periventricular nodular heterotopias (PNH) in HD cases #1 (Fig. 1), #2–3 (Fig. 2), and #8 (Fig. 5c) were morphologically similar to those found in non-HD brains (supplemental Fig. 1) and comprised a population of non-dysmorphic, pyramidal-shaped neurons intermixed with monomorphic glial cells. The neuropil of these nodules lacked gliosis or evidence of neuronal degeneration. One of the striking histologic findings seen in cases #2, #3, #8 (discovery cohort) and #22 (validation cohort) was the distinct contrast between the bland appearance of the heterotopia and the severe gliosis and neuronal loss seen in the adjacent tail of the caudate nucleus (Fig. 2b–d, g–l). However, p62/ HTT inclusions were found in both the heterotopias and in the tail of the caudate nuclei (Fig. 2e–f, m, n). Neurofibrillary tangles were absent in the heterotopias in cases #1–3 and #8 by LH&E and p62 immunostaining. In contrast to heterotopias found in the HD brains, heterotopias of non-HD cases did not show HTT aggregates (*n* = 3, Supplemental Table 1). However, tau-related and synuclein-related aggregates were seen depending on the respective comorbid neurodegenerative conditions as previously described [12, 31]. Accordingly, p62 aggregates reflective of the specific brain proteinopathy were seen in these heterotopias.

**Figure 3:**
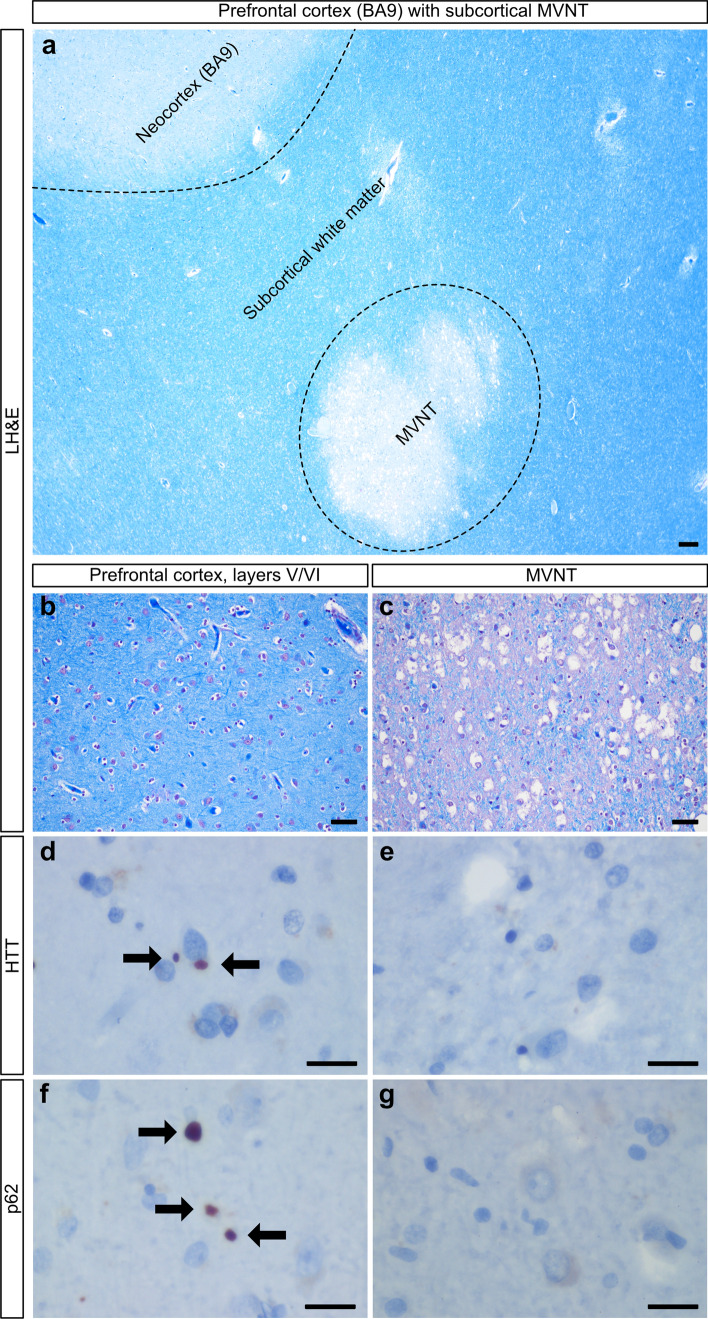
Histopathology of case #5. **a** Photomicrograph of the multinodular vacuolating tumor of the cerebrum (MVNT) that is surrounded by white matter and rests beneath the prefrontal cortex (BA9). **b**, **c** The malformation is vacuolated (**c**) in contrast to the infragranular layers of the nearby prefrontal cortex (**b**). **d**–**g** In contrast to the prefrontal cortex, which has many HTT (**d**) and p62 aggregates (**f**), the MVNT has none (**e**, **g**). Arrows indicate aggregates. Scale bars: **a** 200 µm; **b**, **c** 50 µm; **d**–**g** 20 µm.

The subcortical malformation of case #5 differed with the PNH by morphology and immunohistochemistry. This lobulated mass comprised well-circumscribed foci of immature neurons intermixed with glial cells that were surrounded by a vacuolated neuropil (Fig. 3a, c). A subset of neurons exhibited intracytoplasmic vacuoles. Neither gliosis nor infiltration of the surrounding parenchyma was seen. This morphology was consistent with a multinodular and vacuolating neuronal tumor of the cerebrum (MVNT) [29]. NeuN immunostaining highlighted differentiated neurons within the neighboring cortex; however, the neurons within the malformation were negative (not shown), consistent with the immature neuronal development that is characteristic of MVNT. Moreover, immunohistochemistry failed to detect pathologic HTT or p62 aggregates within this malformation in contrast to that seen in the nearby neocortex (Fig. 3d–g).

**Figure 4:**
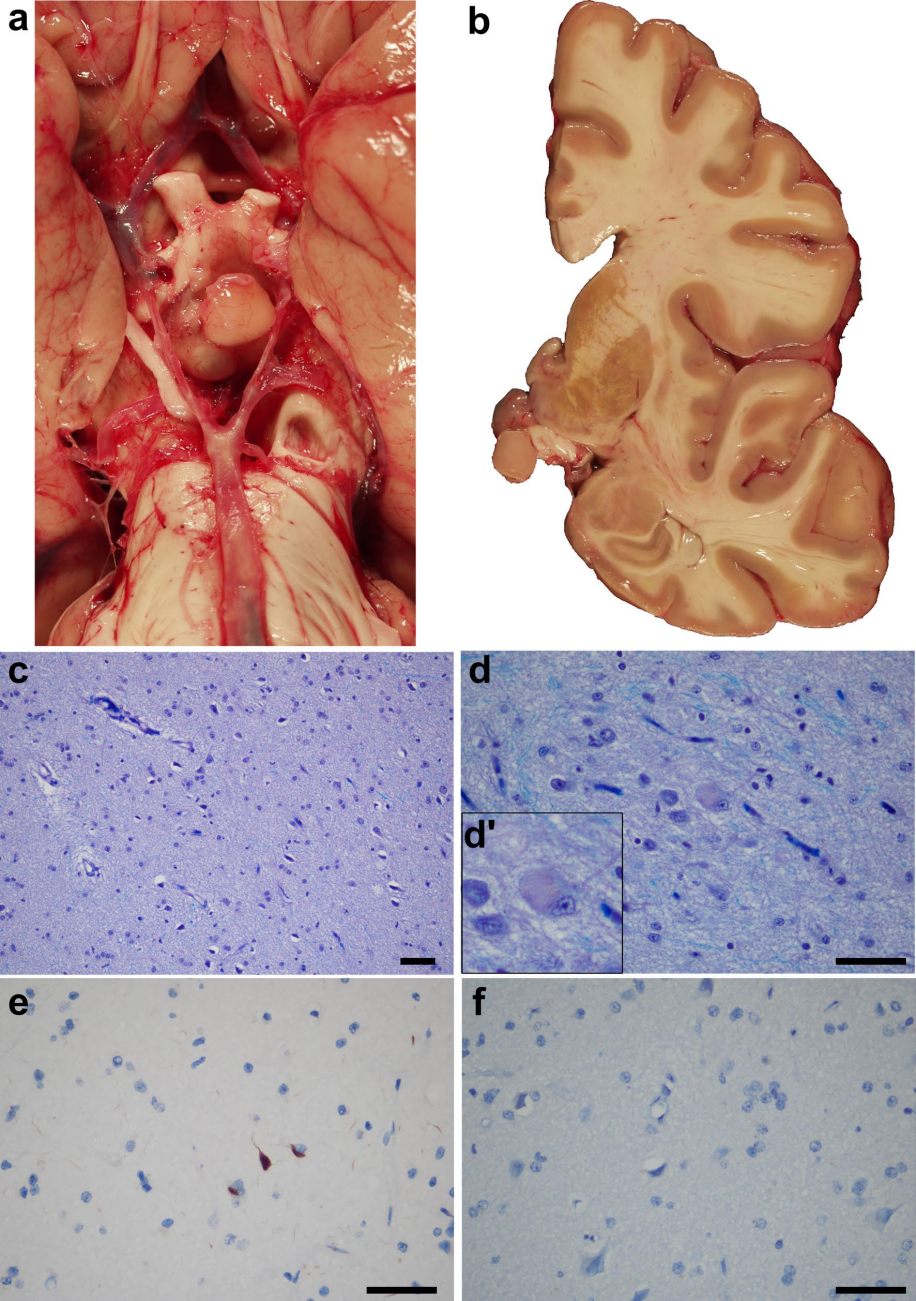
Macroscopic and microscopic photographs of the hypothalamic hamartoma of case #4. **a**, **b** A 0.7 cm, tan, round, firm nodule was noticed in the tuber cinereum next to the hypothalamus in the fresh state. The head of the caudate nucleus shows mild atrophy and retains the normal, medial convexity (HD grade: 2/4). **c**, **d** LH&E-stained sections show a solid glioneuronal mass with haphazardly arranged neurons and intermixed glia (**c**). Occasional neurofibrillary tangles are seen (d, d’ inset). **e**, **f** Whilst a p62 immunostain highlights tangles and neuropil threads within the lesion (**e**), the HTT immunostain is negative for aggregates (**f**). Scale bars: 50 µm.

Case #4 revealed a 0.7 cm firm, tan, solid mass with a smooth surface involving the tuber cinereum (Fig. 4a, b). This nodule was composed of a mixture of glia and non-dysmorphic neurons, a subset of which exhibited neurofibrillary tangles (Fig. 4c, d). The location of this mass and its histomorphology was consistent with that of a hypothalamic hamartoma [15]. Immunostaining against p62 identified neurofibrillary tangles and neuropil threads (Fig. 4e); however, no HTT aggregates were detected by immunohistochemistry (Fig. 4f).

A focal collection of mature, ovoid neurons, and glial cells were present in the subcortical white matter ventral to the dentate nucleus in case #6. Case #7 had a subcortical nodule of mature Purkinje-like neurons and glial cells within the album cerebelli. There were no HTT or p62 inclusions identified within this heterotopia nor in the cerebellar cortex by immunohistochemistry.

**Figure 5:**
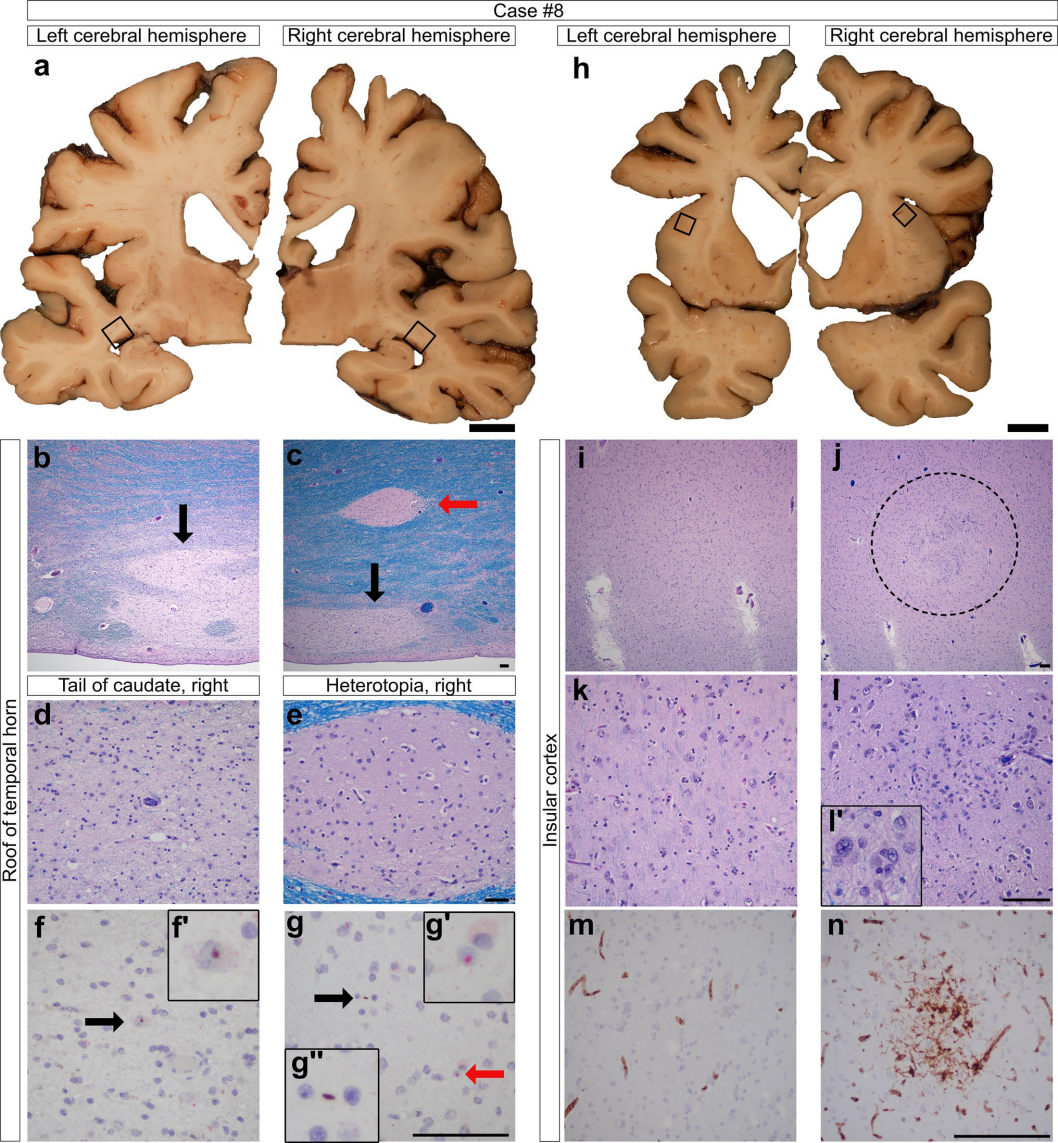
Macroscopic and microscopic photographs of case #8. **a**–**g** At the level of the lateral geniculate body, a PNH (red arrow, **c**) is noted above the atrophic tail of the caudate nucleus (black arrow, **c**), but not in the left cerebral hemisphere (**b**). Similar to the other PNH in HD, the heterotopia (**e**) shows relative sparing of neurodegeneration in comparison to the nearby tail of the caudate nucleus (**d**) and demonstrates p62 (red arrow, g and g’ inset) and HTT aggregates (black arrow, g’’ inset). An intranuclear HTT aggregate is shown in the tail of the caudate nucleus (black arrow, f, f’). **h**–**n** At the level of the nucleus accumbens, an abnormal cluster of cells is noted in the right insular cortex (dashed circle, **j**, **l**). An inset (l’) shows enlarged glial cells and a neuron within the focus. A CD34 immunostain highlights immature cells with bushy processes within this malformation (**n**). The corresponding side on the left insular cortex is provided for comparison (I, **k**, **m**). Note the severe cortical atrophy in this HD brain. Scale bars a, **h**: 1 cm; **b**–**n** 100 µm.

The whole brain of case #8 was fixed and each half was processed according to the same protocol for comparing laterality of the cerebral hemispheres (Fig. 5). A PNH (Fig. 5c, e, g) and a malformation in the insular cortex (Fig. 5j, l, n) were found in the right cerebral hemisphere. The 1 × 1 mm circumscribed insular malformation appeared as a focal disorganization of the laminar architecture in the infragranular layers of the cortex (Fig. 5j). This malformation was composed of a collection of haphazard, enlarged, pleomorphic glial cells spread amongst closely apposed neurons with hyperchromatic nuclei. Neither neuronal dysmorphism nor balloon cells were evident. CD34 immunostaining demonstrated a cluster of cells with bushy processes (Fig. 5n), confirming cellular immaturity within this malformation. HTT and p62 aggregates were not seen in the anomaly; however, they were found in the nearby neocortex. Altogether, the histomorphology appeared most consistent with that of a glial microhamartoma (alternatively known as a glial heterotopia) that has been described in the context of neurofibromatosis type 2 (NF2) [53, 65]. This individual did not have a history of NF2 nor other stigmata of the syndrome at post-mortem examination.

## Discussion

By examining two large, independent autopsy cohorts, we demonstrate an increased frequency of malformations in the brains of individuals with HD and thus provide further evidence that pathogenic trinucleotide repeat expansions of the *HTT* gene may impact neurodevelopment. The majority of these malformations in HD were PNH (10 of 15) and appeared asymmetric at macroscopic examination, suggesting that these may have developed secondary to a post-zygotic somatic mutation or another developmental disruption [48]. Malformations were found in patients with varied disease severity and were individuals with adult-onset of motor signs that had *HTT* repeat expansions between 40 and 52 CAG repeats. In both of our HD cohorts, women more frequently harbored malformations than men, thereby suggesting the possible presence of sexual dimorphism in specific developmental contexts, such as malformations within the rostral neuraxis.

These malformations would have formed early during neurodevelopment and before the clinical onset of HD. The increased occurrence of malformations in HD brains therefore reflects a focal disturbance in usual brain development caused by *HTT* gene expansion rather than as a consequence of clinical disease. However, the pathogenic *HTT*-related deleterious process may be occurring earlier and closer to the timing of neurodevelopmental events than previously envisaged. Well documented are the subtle clinicopathologic changes that occur before the clinical disease onset; premanifest carriers with pathogenic *HTT* gene expansion show striatal atrophy, cortical thinning, focally increased oligodendrocyte densities, and demonstrate cognitive and behavioral changes that can occur up to several decades prior to the onset of motor signs [19, 26, 46, 49, 58].

We found 5 types of developmental malformations that were hitherto not associated with HD and PNH were the most common. Many molecular mechanisms regulating heterotopia formation are recognized, and the etiology includes both genetic aberrations and epigenetic phenomena. Filamin A (*FLNA*) mutations are the most well-known cause of PNH. FLNA is an actin-binding protein that is cell autonomous in neuronal migration by its expression in cortical precursors. Inheritance of an X-linked mutation of the *FLNA* gene causes bilateral PNH in females or lethality in males, whereas missense mutations that functionally result in hypomorphic alleles lead to viable males and focal PNH in females [23, 44, 55]. Such a missense mutation in *FLNA* could produce the focal PNH in the HD women in this study (cases #1–3, 8, 22, 24, 26–27). In MVNT, a malformation that was identified in HD case #5, mutations in genes involved in the MAP kinase pathway have been reported [50]. This increased frequency of malformations in HD may be caused by disease-related genomic instability as a result of *HTT* gene expansion [1, 33, 34, 43, 57]. The rate of somatic mutation is high during neurogenesis at approximately 5.1 single-nucleotide variants per progenitor per day and pathogenic mutations during this period may cause malformations [7, 17]. HTT is implicated in DNA repair and, if this is partially defective through *HTT* gene expansion, this could further increase the frequency of post-zygotic somatic mutations in neuronal precursors and lead to an increased occurrence of malformations in GEC [22, 24, 30, 37, 39, 40, 51].

However, loss-of-function of HTT may also play a role. Clearly, the developmental biology of HTT species, its modifications, protein interactors, and molecular pathway effectors are critical for establishing the dynamic scaffolds necessary for cortical organization (summarized in [54]). Supporting the loss-of-function hypothesis, the murine *HTT* hypomorph model investigated by Arteaga-Bracho and colleagues developed subpallial heterotopias and, later in life, striatal neurodegeneration [5]. Moreover, Barnat et al*.* have demonstrated abnormal localization of HTT and junctional complexes within neural progenitor cells at 13 week gestational age in human fetal GEC [10]. Such junctions are crucial for apicobasal polarity within the neuroepithelium and for cellular cohesion. If perturbed, this could affect neuronal migration and also lead to heterotopia formation [21, 36, 56, 61].

The variety and differing pathogenesis of these malformations provides further insight into the neurodegenerative aspects of HD. The relative sparing of neurodegeneration within the PNH in the roof of the temporal horn was in stark contrast to the nearby atrophic tail of the caudate nucleus and emphasizes the selective vulnerability of cell types and connections in HD, particularly the neostriatal medium spiny neurons. Furthermore, the presence of HTT/p62 aggregates in PNH is similar to that of the HD neocortex. This occurrence is expected as these heterotopic neurons would have been destined for the cortex which is usually rich in aggregates. Moreover, this would also explain why definite neuronal loss is not obvious in the PNH compared to the adjacent neostriatum; although the HD neocortex undergoes neuronal loss, it occurs to a lesser extent than that seen in the neostriatum.

In contrast to the PNH, aggregates were not found within the MVNT, hypothalamic hamartoma, cerebellar heterotopia, and glial microhamartoma of HD brains. This likely reflects the different neuronal subtypes within the respective malformation and/or the different stages of neuronal development within these anomalies. For example, the vacuolated neurons of MVNT usually express OLIG2 but not NeuN, which has led to the hypothesis that these cells may be the sequela of interrupted development of neural progenitor cells [59]. Furthermore, the presence of CD34, a stem cell marker expressed during neurulation, in the glial microhamartoma (case #8) indicates cellular immaturity within this malformation and might explain why aggregates do not occur within these cells in contrast to those of the adjacent cortex [13].

The reduced detection of malformations in HD in the validation cohort than the discovery cohort is to some extent due to the differential processing methods between the two centers and the cumulative experience over time. In the discovery cohort at the NYBB, trained pathologists examine the macroscopic slices of both halves of the brain, whereas only one fixed half brain was usually examined by a neuropathologist in the validation cohort [62]. Furthermore, the standardized series of blocks obtained for the microscopic examination of most half brains in the validation cohort utilized only 14 blocks, whereas four additional blocks were taken in the discovery cohort. Therefore, this greater sampling and professional experience in the discovery cohort increases the probability of detecting malformations.

There are limitations to this study. Given that HD is heritable, it is possible that some of the brains with malformations are related and thus share common genes that predispose for such anomalies, thereby biasing the HD cohort. Review of the medical charts did not reveal any association of heterotopias between siblings. Birth and maternal histories were unavailable. Furthermore, brains received at a brain bank may be subject to referral bias, such that patients with diagnostically interesting or unusual presentations may be encouraged to consent for brain donation. This is unavoidable, although using a validation cohort of a much larger size based from a different region of the United States of America, we have aimed to overcome this potential bias. Finally, we cannot provide a potential biological mechanism by employing these formalin-fixed tissue samples.

In conclusion, we document an increased frequency of neurodevelopmental malformations in the HD brain compared to non-HD brains in the largest neuropathologic series of HD to date that spans a period of nearly 40 years. We further demonstrated that cortical malformations of the cerebrum that contain mature neurons were susceptible to HTT/p62 aggregation, whereas those with immature neuronal populations lacked such aggregates. Whether more subtle neurodevelopmental abnormalities are occurring in the HD brain is conceivable and merits further study.

## Supplementary Information

Below is the link to the electronic supplementary material.

Supplementary file1 (DOCX 6595 KB)

## References

[1] (2019) CAG Repeat Not Polyglutamine Length Determines Timing of Huntington's Disease Onset. Cell 178:887–900.e814. 10.1016/j.cell.2019.06.036 10.1016/j.cell.2019.06.036 PMC6700281 31398342

[2] (2018) CD34 antibody- QBEND/10 https://www.bio-rad-antibodies.com/monoclonal/human-cd34-antibody-qbend-10-mca547.html?f=purified&JSESSIONID_STERLING=058B98689A299BF23B7F87D8DB79FE92.ecommerce2&evCntryLang=AT-de&cntry=AT&thirdPartyCookieEnabled=true

[3] (2017) Developmental alterations in Huntington's disease neural cells and pharmacological rescue in cells and mice. Nat Neurosci 20: 648-660. 10.1038/nn.4532 10.1038/nn.4532 PMC5610046 28319609

[4] (2018) Periventricular heterotopia https://medlineplus.gov/genetics/condition/periventricular-heterotopia/. Accessed 9 Jul 2020

[5] Arteaga-Bracho EE, Gulinello M, Winchester ML, Pichamoorthy N, Petronglo JR, Zambrano AD, Inocencio J, De Jesus CD, Louie JO, Gokhan S (2016). Postnatal and adult consequences of loss of huntingtin during development: implications for Huntington's disease. Neurobiol Dis.

[6] Auerbach W, Hurlbert MS, Hilditch-Maguire P, Wadghiri YZ, Wheeler VC, Cohen SI, Joyner AL, MacDonald ME, Turnbull DH (2001). The HD mutation causes progressive lethal neurological disease in mice expressing reduced levels of huntingtin. Hum Mol Genet.

[7] Bae T, Tomasini L, Mariani J, Zhou B, Roychowdhury T, Franjic D, Pletikos M, Pattni R, Chen B-J, Venturini E (2018). Different mutational rates and mechanisms in human cells at pregastrulation and neurogenesis. Science (New York, NY).

[8] Barkovich A, Kjos B (1992). Gray matter heterotopias: MR characteristics and correlation with developmental and neurologic manifestations. Radiology.

[9] Barkovich AJ, Guerrini R, Kuzniecky RI, Jackson GD, Dobyns WB (2012). A developmental and genetic classification for malformations of cortical development: update 2012. Brain.

[10] 10Barnat M, Capizzi M, Aparicio E, Boluda S, Wennagel D, Kacher R, Kassem R, Lenoir S, Agasse F, Braz BY (2020) Huntington’s disease alters human neurodevelopment. Science (New York, NY) 10.1126/science.aax3338 PMC7859879 32675289

[11] Bhide PG, Day M, Sapp E, Schwarz C, Sheth A, Kim J, Young AB, Penney J, Golden J, Aronin N (1996). Expression of normal and mutant huntingtin in the developing brain. J Neurosci.

[12] Bieniek KF, Dickson DW (2015). Concurrent neurodegenerative pathologies in periventricular nodular heterotopia. Acta Neuropathol.

[13] Blümcke I, Giencke K, Wardelmann E, Beyenburg S, Kral T, Sarioglu N, Pietsch T, Wolf HK, Schramm J, Elger CE (1999). The CD34 epitope is expressed in neoplastic and malformative lesions associated with chronic, focal epilepsies. Acta Neuropathol.

[14] 14Brodmann K (1909) Vergleichende Lokalisationslehre der Grosshirnrinde in ihren Prinzipien dargestellt auf Grund des Zellenbaues. Barth, City

[15] Coons SW, Rekate HL, Prenger EC, Wang N, Drees C, Ng Y-t, Chung SS, Kerrigan JF (2007). The histopathology of hypothalamic hamartomas: study of 57 cases. J Neuropathol Exp Neurol.

[16] Croce KR, Yamamoto A (2019). A role for autophagy in Huntington's disease. Neurobiol Dis.

[17] 17D’Gama AM, Walsh CA (2018) Somatic mosaicism and neurodevelopmental disease. Nat Neurosci 21:1504–1514 10.1038/s41593-018-0257-3 30349109

[18] Dehay B, Weber C, Trottier Y, Bertolotti A (2007). Mapping of the epitope of monoclonal antibody 2B4 to the proline-rich region of human Huntingtin, a region critical for aggregation and toxicity. Biotechnol J.

[29] Duff K, Paulsen JS, Beglinger LJ, Langbehn DR, Stout JC, Group P-HIotHS (2007) Psychiatric symptoms in Huntington’s disease before diagnosis: the predict-HD study. Biol Psychiatry 62:1341–1346 10.1016/j.biopsych.2006.11.034 17481592

[20] Duyao MP, Auerbach AB, Ryan A, Persichetti F, Barnes GT, McNeil SM, Ge P, Vonsattel JP, Gusella JF, Joyner AL (1995). Inactivation of the mouse Huntington's disease gene homolog Hdh. Science (New York, NY).

[21] Ferland RJ, Batiz LF, Neal J, Lian G, Bundock E, Lu J, Hsiao Y-C, Diamond R, Mei D, Banham AH (2009). Disruption of neural progenitors along the ventricular and subventricular zones in periventricular heterotopia. Human Mol Genet.

[22] Ferlazzo ML, Sonzogni L, Granzotto A, Bodgi L, Lartin O, Devic C, Vogin G, Pereira S, Foray N (2014). Mutations of the Huntington's disease protein impact on the ATM-dependent signaling and repair pathways of the radiation-induced DNA double-strand breaks: corrective effect of statins and bisphosphonates. Mol Neurobiol.

[23] Fox JW, Lamperti ED, Ekşioğlu YZ, Hong SE, Feng Y, Graham DA, Scheffer IE, Dobyns WB, Hirsch BA, Radtke RA (1998). Mutations in filamin 1 prevent migration of cerebral cortical neurons in human periventricular heterotopia. Neuron.

[24] Gao R, Chakraborty A, Geater C, Pradhan S, Gordon KL, Snowden J, Yuan S, Dickey AS, Choudhary S, Ashizawa T (2019). Mutant huntingtin impairs PNKP and ATXN3, disrupting DNA repair and transcription. Elife.

[25] Godin JD, Colombo K, Molina-Calavita M, Keryer G, Zala D, Charrin BC, Dietrich P, Volvert ML, Guillemot F, Dragatsis I (2010). Huntingtin is required for mitotic spindle orientation and mammalian neurogenesis. Neuron.

[26] Gómez-Tortosa E, MacDonald ME, Friend JC, Taylor SA, Weiler LJ, Cupples LA, Srinidhi J, Gusella JF, Bird ED, Vonsattel JP (2001). Quantitative neuropathological changes in presymptomatic Huntington's disease. Ann Neurol.

[27] Herndon ES, Hladik CL, Shang P, Burns DK, Raisanen J, White CL (2009). Neuroanatomic profile of polyglutamine immunoreactivity in Huntington disease brains. J Neuropathol Exp Neurol.

[28] Hickman RA, Flowers XE, Wisniewski T (2020). Primary age-related tauopathy (PART): addressing the spectrum of neuronal tauopathic changes in the aging brain. Curr Neurol Neurosci Rep.

[29] Huse JT, Edgar M, Halliday J, Mikolaenko I, Lavi E, Rosenblum MK (2013). Multinodular and vacuolating neuronal tumors of the cerebrum: 10 cases of a distinctive seizure-associated lesion. Brain Pathol.

[30] Jamuar SS, Lam A-TN, Kircher M, D’Gama AM, Wang J, Barry BJ, Zhang X, Hill RS, Partlow JN, Rozzo A (2014). Somatic mutations in cerebral cortical malformations. N Engl J Med.

[31] Joseph JT (1997). Periventricular heterotopias display cortical degenerative neuropathology. Neurology.

[32] Keller CE, Del Amaya MP, Cortes EP, Mancevska K, Vonsattel JPG (2008). Electronic tracking of human brain samples for research. Cell Tissue Banking.

[33] Kennedy L, Evans E, Chen CM, Craven L, Detloff PJ, Ennis M, Shelbourne PF (2003). Dramatic tissue-specific mutation length increases are an early molecular event in Huntington disease pathogenesis. Hum Mol Genet.

[34] Kennedy L, Shelbourne PF (2000). Dramatic mutation instability in HD mouse striatum: does polyglutamine load contribute to cell-specific vulnerability in Huntington's disease?. Hum Mol Genet.

[35] Lee JK, Mathews K, Schlaggar B, Perlmutter J, Paulsen JS, Epping E, Burmeister L, Nopoulos P (2012). Measures of growth in children at risk for Huntington disease. Neurology.

[36] Lian G, Sheen VL (2015). Cytoskeletal proteins in cortical development and disease: actin associated proteins in periventricular heterotopia. Front Cell Neurosci.

[37] Lodato MA, Rodin RE, Bohrson CL, Coulter ME, Barton AR, Kwon M, Sherman MA, Vitzthum CM, Luquette LJ, Yandava CN (2018). Aging and neurodegeneration are associated with increased mutations in single human neurons. Science (New York, NY).

[38] MacDonald ME, Ambrose CM, Duyao MP, Myers RH, Lin C, Srinidhi L, Barnes G, Taylor SA, James M, Groot N (1993). A novel gene containing a trinucleotide repeat that is expanded and unstable on Huntington's disease chromosomes. Cell.

[39] Maiuri T, Bowie LE, Truant R (2019). DNA repair signaling of huntingtin: the next link between late-onset neurodegenerative disease and oxidative DNA damage. DNA Cell Biol.

[40] Maiuri T, Mocle AJ, Hung CL, Xia J, van Roon-Mom WM, Truant R (2017). Huntingtin is a scaffolding protein in the ATM oxidative DNA damage response complex. Hum Mol Genet.

[41] Marder K, Mehler MF (2012). Development and neurodegeneration: turning HD pathogenesis on its head. Neurology.

[42] Mehler MF, Petronglo JR, Arteaga-Bracho EE, Gulinello ME, Winchester ML, Pichamoorthy N, Young SK, DeJesus CD, Ishtiaq H, Gokhan S (2019). Loss-of-Huntingtin in medial and lateral ganglionic lineages differentially disrupts regional interneuron and projection neuron subtypes and promotes Huntington's disease-associated behavioral, cellular, and pathological hallmarks. J Neurosci.

[43] Mouro Pinto R, Arning L, Giordano JV, Razghandi P, Andrew MA, Gillis T, Correia K, Mysore JS, Grote Urtubey D-M, Parwez CR (2020). Patterns of CAG repeat instability in the central nervous system and periphery in Huntington’s disease and in spinocerebellar ataxia type 1. Human Mol Genet.

[44] Nagano T, Yoneda T, Hatanaka Y, Kubota C, Murakami F, Sato M (2002). Filamin A-interacting protein (FILIP) regulates cortical cell migration out of the ventricular zone. Nat Cell Biol.

[45] Nasir J, Floresco SB, O'Kusky JR, Diewert VM, Richman JM, Zeisler J, Borowski A, Marth JD, Phillips AG, Hayden MR (1995). Targeted disruption of the Huntington's disease gene results in embryonic lethality and behavioral and morphological changes in heterozygotes. Cell.

[46] Nopoulos P, Magnotta VA, Mikos A, Paulson H, Andreasen NC, Paulsen JS (2007). Morphology of the cerebral cortex in preclinical Huntington's disease. Am J Psychiatry.

[47] Nopoulos PC, Aylward EH, Ross CA, Mills JA, Langbehn DR, Johnson HJ, Magnotta VA, Pierson RK, Beglinger LJ, Nance MA (2011). Smaller intracranial volume in prodromal Huntington's disease: evidence for abnormal neurodevelopment. Brain.

[48] Oegema R, Barkovich AJ, Mancini GMS, Guerrini R, Dobyns WB (2019) Subcortical heterotopic gray matter brain malformations: Classification study of 107 individuals. Neurology. 10.1212/wnl.0000000000008200 10.1212/WNL.0000000000008200 PMC6814414 31484711

[49] Paulsen JS, Nopoulos PC, Aylward E, Ross CA, Johnson H, Magnotta VA, Juhl A, Pierson RK, Mills J, Langbehn D (2010). Striatal and white matter predictors of estimated diagnosis for Huntington disease. Brain Res Bull.

[50] Pekmezci M, Stevers M, Phillips JJ, Van Ziffle J, Bastian BC, Tsankova NM, Kleinschmidt-DeMasters BK, Rosenblum MK, Tihan T, Perry A (2018). Multinodular and vacuolating neuronal tumor of the cerebrum is a clonal neoplasm defined by genetic alterations that activate the MAP kinase signaling pathway. Acta Neuropathol.

[51] Poduri A, Evrony GD, Cai X, Walsh CA (2013). Somatic mutation, genomic variation, and neurological disease. Science (New York, NY).

[52] Raymond A, Fish D, Sisodiya S, Alsanjari N, Stevens J, Shorvon S (1995). Abnormalities of gyration, heterotopias, tuberous sclerosis, focal cortical dysplasia, microdysgenesis, dysembryoplastic neuroepithelial tumour and dysgenesis of the archicortex in epilepsy: clinical, EEG and neuroimaging features in 100 adult patients. Brain.

[53] 53Russell DS, Rubinstein LJ (1989) Dysplastic lesions of the central nervous system. Pathology of tumours of the nervous system. William s & Wilkins, City, pp 776–781

[54] Saudou F, Humbert S (2016). The biology of huntingtin. Neuron.

[55] Sheen VL, Dixon PH, Fox JW, Hong SE, Kinton L, Sisodiya SM, Duncan JS, Dubeau F, Scheffer IE, Schachter SC (2001). Mutations in the X-linked filamin 1 gene cause periventricular nodular heterotopia in males as well as in females. Hum Mol Genet.

[56] Sheen VL, Ganesh VS, Topcu M, Sebire G, Bodell A, Hill RS, Grant PE, Shugart YY, Imitola J, Khoury SJ (2004). Mutations in ARFGEF2 implicate vesicle trafficking in neural progenitor proliferation and migration in the human cerebral cortex. Nat Genet.

[57] Shelbourne PF, Keller-McGandy C, Bi WL, Yoon SR, Dubeau L, Veitch NJ, Vonsattel JP, Wexler NS, Arnheim N, Augood SJ (2007). Triplet repeat mutation length gains correlate with cell-type specific vulnerability in Huntington disease brain. Hum Mol Genet.

[58] Solomon AC, Stout JC, Johnson SA, Langbehn DR, Aylward EH, Brandt J, Ross CA, Beglinger L, Hayden MR, Kieburtz K (2007). Verbal episodic memory declines prior to diagnosis in Huntington's disease. Neuropsychologia.

[59] Thom M, Liu J, Bongaarts A, Reinten RJ, Paradiso B, Jäger HR, Reeves C, Somani A, An S, Marsdon D (2018). Multinodular and vacuolating neuronal tumors in epilepsy: dysplasia or neoplasia?. Brain Pathol.

[60] van der Plas E, Langbehn DR, Conrad AL, Koscik TR, Tereshchenko A, Epping EA, Magnotta VA, Nopoulos PC (2019). Abnormal brain development in child and adolescent carriers of mutant huntingtin. Neurology.

[61] Veeraval L, O'Leary CJ, Cooper HM (2020). Adherens junctions: guardians of cortical development. Front Cell Dev Biol.

[62] Vonsattel JP, Del Amaya MP, Keller CE (2008). Twenty-first century brain banking. Processing brains for research: the Columbia University methods. Acta Neuropathol.

[63] Vonsattel JP, Myers RH, Stevens TJ, Ferrante RJ, Bird ED, Richardson EP (1985). Neuropathological classification of Huntington's disease. J Neuropathol Exp Neurol.

[64] White JK, Auerbach W, Duyao MP, Vonsattel JP, Gusella JF, Joyner AL, MacDonald ME (1997). Huntingtin is required for neurogenesis and is not impaired by the Huntington's disease CAG expansion. Nat Genet.

[65] Wiestler OD, von Siebenthal K, Schmitt HP, Feiden W, Kleihues P (1989). Distribution and immunoreactivity of cerebral micro-hamartomas in bilateral acoustic neurofibromatosis (neurofibromatosis 2). Acta Neuropathol.

