# Supplementary material for: Developmental malformations in Huntington disease: neuropathologic evidence of focal neuronal migration defects in a subset of adult brains
Source: Acta Neuropathol. 2021 Jan 30;141(3):399–413. doi: 10.1007/s00401-021-02269-4 (PMC7882590; doi:10.1007/s00401-021-02269-4)
Supplement: Supplementary file 1 — Supplementary file1 (DOCX 6595 KB) [file 401_2021_2269_MOESM1_ESM.docx]

Supplementary data

**Supplemental table 1: Clinical characteristics of HD and non-HD cases**

| **Discovery cohort** | | | |
| --- | --- | --- | --- |
| **Characteristic** | **HD** | **Non-HD** | ***P* value** |
| Median age at death (IQR) | 56 (21) | 80 (17) | <0.0001 |
| Sex (% female) | 46.9 | 49.7 | 0.59 |
| Median CAG (range) | 45 (38-105) | N/A | - |
| Total number of brains (participants) examined | 130 | 1600 | - |
| **Validation cohort** | | | |
| **Characteristic** | **HD** | **Non-HD** | ***P* value** |
| Median age at death (IQR) | 61 (22) | 76 (17) | <0.0001 |
| Sex (% female) | 48.1 | 47.6 | 0.79 |
| Total number of half brains | 720 | 1989 | - |
| Total number of participants | 619 | 1700 | - |


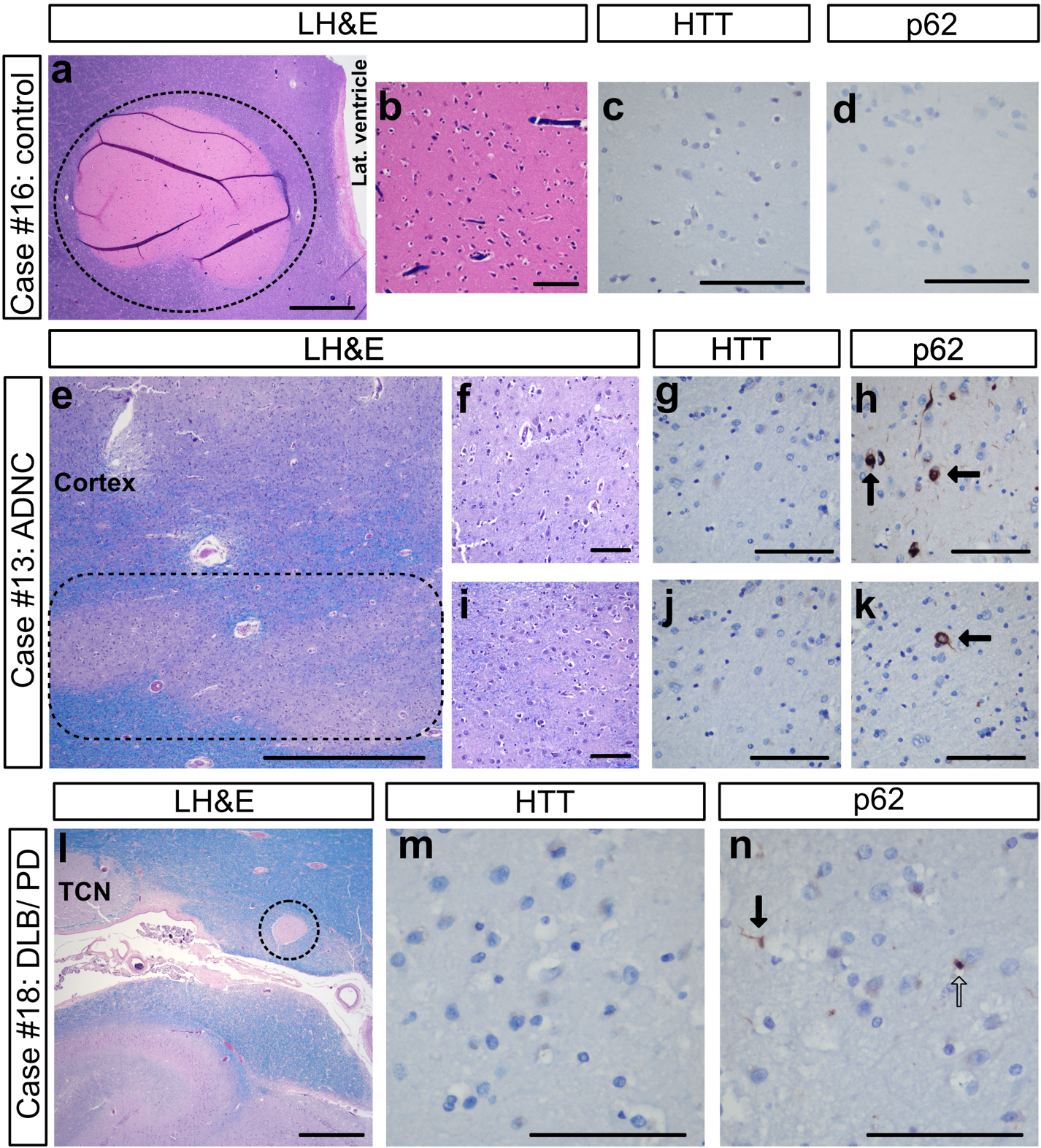


**Supplemental figure 1: Histologic and immunohistochemical staining characteristics of malformations in non-HD tissues (control, ADNC, DLB/PD). No aggregated HTT are found in these heterotopia. a-d: The periventricular nodular heterotopia of case #16 comprised well-circumscribed gray matter set within white matter (dashed outline, a). b: The heterotopia was composed of mature neurons and glia. c-d: Neither HTT (c) or p62 (d) aggregates were identified in this heterotopia. e-k: The subcortical band heterotopia (dashed outline, e) was separated from the overlying temporal cortex by a thin rim of myelinated white matter (e). Both the cortex (f) and subcortical band heterotopia (i) harbor mature neurons and glia and lack HTT aggregates (g, j). However, p62-labeled neuropil threads and tangles are found in both the cortex (arrows, h) and heterotopia (arrows, k) and reflect the tauopathic burden in both components (not shown). l-n: In case 18, a periventricular nodular heterotopia (dashed outline, l) is found above the hippocampus and medial to the tail of the caudate nucleus (TCN). m-n: HTT aggregates are not seen in this heterotopia (m), however, p62-labeled aggregates are identified (arrows, n) that likely reflect Lewy neurites (solid arrow, n) and Lewy bodies (open arrow, n) seen in the heterotopia (not shown). Scale bars: a, e, l: 1mm; b-d, f-k, m-n: 100 µm.**
